# Cloning and Expression Analysis of Litchi (*Litchi Chinensis* Sonn.) Polyphenol Oxidase Gene and Relationship with Postharvest Pericarp Browning

**DOI:** 10.1371/journal.pone.0093982

**Published:** 2014-04-24

**Authors:** Jiabao Wang, Baohua Liu, Qian Xiao, Huanling Li, Jinhua Sun

**Affiliations:** Environment and Plant Protection Research Institute, Chinese Academy of Tropical Agriculture Sciences, Haikou, Hainan, China; Universidade Federal do Rio Grande do Sul, Brazil

## Abstract

Polyphenol oxidase (PPO) plays a key role in the postharvest pericarp browning of litchi fruit, but its underlying mechanism remains unclear. In this study, we cloned the litchi PPO gene (*LcPPO*, JF926153), and described its expression patterns. The *LcPPO* cDNA sequence was 2120 bps in length with an open reading frame (ORF) of 1800 bps. The ORF encoded a polypeptide with 599 amino acid residues, sharing high similarities with other plant PPO. The DNA sequence of the ORF contained a 215-bp intron. After carrying out quantitative RT-PCR, we proved that the *LcPPO* expression was tissue-specific, exhibiting the highest level in the flower and leaf. In the pericarp of newly-harvested litchi fruits, the *LcPPO* expression level was relatively high compared with developing fruits. Regardless of the litchi cultivar and treatment conditions, the *LcPPO* expression level and the PPO activity in pericarp of postharvest fruits exhibited the similar variations. When the fruits were stored at room temperature without packaging, all the pericarp browning index, PPO activity and the *LcPPO* expression level of litchi pericarps were reaching the highest in Nandaowuhe (the most rapid browning cultivar), but the lowest in Ziniangxi (the slowest browning cultivar) within 2 d postharvest. Preserving the fruits of Feizixiao in 0.2-μm plastic bag at room temperature would decrease the rate of pericarp water loss, delay the pericarp browning, and also cause the reduction of the pericarp PPO activity and *LcPPO* expression level within 3 d postharvest. In addition, postharvest storage of Feizixiao fruit stored at 4°C delayed the pericarp browning while decreasing the pericarp PPO activity and *LcPPO* expression level within 2 d after harvest. Thus, we concluded that the up-regulation of *LcPPO* expression in pericarp at early stage of postharvest storage likely enhanced the PPO activity and further accelerated the postharvest pericarp browning of litchi fruit.

## Introduction

Polyphenol oxidases (PPOs) are a group of metalloproteinases encoded by nuclear genes and capable of binding copper. The PPO family include monophenol oxidase (tyrosinase, EC 1.14.18.1), diphenol oxidase (catechol oxidase, EC 1.10.3.1), and laccase (EC 1.10.3.2). In general, catechol oxidase and laccase are collectively referred to as PPOs. PPOs can catalyze oxidation of the monophenolic hydroxyl group and dihydric phenol to *o*-diphenol and *o*-quinone, respectively. Quinones can self-polymerize in the plant body or react with amino acids and proteins in the cell, leading to the formation of black or brown substances and the browning of plant tissue. This reaction is considered to be the main reason for the postharvest browning of fruits and vegetables. [1]–[3].

Litchi (*Litchi Chinensis* Sonn.) is one of the important fruits in South China. In the year of 2010, the planting area of litchi was approximately 552,000 hectares and the fruit yield was 1.77 million tons. However, the rapid postharvest browning of pericarp reduced the commodity value of the fruit and limited the expansion of the litchi trade, and was considered as the most important limitation to the continued development of the litchi industry [4]–[5]. Li and his colleges first proposed that litchi pericarp browning is an enzymatic browning process caused by PPO activity [6]. Furthermore, several reports demonstrated that the partially purified PPO enzyme could only oxidize polyphenols containing the *ortho*- hydroxyl group, suggests that the enzyme lacked cresolase and laccase activities and was a diphenol oxidase [7]–[10]. When a reaction was carried out with the natural substrate, the phenols isolated from litchi pericarp, and partially-purified litchi pericarp PPO, the brown substance(s) formed [7], [11]. Further research indicated the pericarp contains a variety of phenols; most of them could be oxidized by PPO [12]. The (-)-epicatechin [13] and procyanidin A2 [14] occurring at relatively high levels in pericarp are the direct substrates of PPO. Additionally, PPO oxidizes anthocyanin in the presence of polyphenol, resulting in the formation of brown substances [10]. To take together, these findings confirm that pericarp contains the substrates of PPO. The distribution range of PPO activity in pericarp is generally consistent with that of phenols. Both of them are present in the epidermis and sclerenchyma cells of exocarp and some of the cells of the mesocarp and endocarp [15], [16]. The PPO activity in the easily-browned exocarp is higher than the slightly-browned mesocarp and endocarp [16]. During the postharvest aging process, the pericarp PPO activity diffuses in proportion with the browning site, consistent with the browning process [15]. When the PPO activity is inhibited by fruit treatments such as cold storage, chemical preservative, and coating, the pericarp browning process is also slowed down [5], [17]. In contrast, when the PPO activity is enhanced by fungal infection [18] or ethylene treatment [19], the pericarp browning is accelerated. Therefore, phenol oxidation catalyzed by PPO in pericarp may be a direct cause of pericarp browning, and reduction of the PPO activity in pericarp is expected to inhibit pericarp browning.

Inhibition of PPO gene expression in plant tissues reduces the PPO activityand tissue browning. For example, the PPO activity and browning potential are reduced in apple caulicles and calluses, and this process is associated with the expression of PPO antisense RNA [20]. Expression of potato PPO antisense RNA in tomato reduces the levels of tomato PPO gene expression and PPO activity, further reduces the capacity for oxidation of the caffeic acid in tomato leaves by approximately 40-folds [21]. In addition, expression of PPO antisense RNA in “Yali” pear can cause the decreases of the PPO activity in pear leaves [22]. Initially, we hypothesize that specific inhibition of PPO expression in litchi pericarp would suppress postharvest pericarp browning. In the present study, the litchi PPO gene, *LcPPO*, was cloned in order to investigate the relationships among *LcPPO* expression, the pericarp PPO activity and the postharvest browning process. Our paper proved solid data which support that *LcPPO* expression is up-regulated and the PPO activity is enhanced at an early stage of postharvest storage, possibly leading to the acceleration of pericarp browning. Our conclusion can facilitate the further elucidation of the mechanism of litchi pericarp browning which can be applied to the breeding of long-storage litchi cultivars by using bio-engineering techniques.

## Materials and Methods

### Litchi fruits

In the year of 2010, litchi fruits of three different cultivars, Feizhixiao, Ziniangxi, and Nandaowuhe were obtained from a test base of the Chinese Academy of Tropical Agriculture Sciences. Some young fruits were collected every week from each litchi cultivar after the fruit size reached 1 cm in diameter to the day on May 14^th^ of the year, when most of Feizixiao fruits reached 80% maturity and were harvested. Fruits with the same maturity of Ziniangxi and Nandaowuhe were harvested on June 7^th^ of 2010. All fruits were transported to the laboratory within 3 h after the harvest and sorted immediately by selecting those of equivalent maturity, without pests, diseases, and mechanical injuries. The selected fruits were dipped in 1 g · L^−1^ iprodione for 3 min and then air-dried naturally for approximately 10 min prior to the postharvest handling.

The fruits (20 fruits per group) were processed in triplicate groups. The pericarp was peeled from young fruits of each cultivar; the pericarp, pulp, and seeds were picked up from newly-harvested 80%-mature fruits of Feizixiao. Semi-lignified spring shoots and their young leaves, unopened flower buds, and the absorbing roots of seedlings of Feizixiao were collected respectively. The pericarp was peeled from fruits of Feizixiao, Ziniangxi, and Nandaowuhe cultivars after different treatments. All tissue samples were immediately frozen in liquid nitrogen (LN2) and stored at −70°C till use.

### Postharvest handling and sampling

The level of pericarp browning and the PPO activity as well as the *LcPPO* expression in different litchi cultivars was evaluated in the laboratory. A single layer of fruits of Feizixiao, Ziniangxi, and Nandaowuhe cultivars were arranged in preserving containers (30 cm×20 cm×10 cm, 20 fruits each) without squeezing. Afterwards, the containers were covered with lids while maintaining a 1-cm gap. The fruits were stored at 25°C and the specimens were taken out daily from the day of the harvest (day 0) to the day the pericarp turned completely brown.

The effect of pericarp water loss on the PPO activity and *LcPPO* expression were evaluated using the following method. At the beginning, the fruits of Feizixiao were placed in 0.2-μm plastic bags (20 fruits each) and then stored at 25°C. Specimens were taken out after the day of the harvest (day 0) and days 1, 2, 3, 5, and 7 postharvest. The fruits of Feizixiao from the previous experiment were used as control.

The effect of low temperature on the PPO activity and *LcPPO* expression of different litchi cultivars was assessed. The fruits of Feizixiao were placed in preserving containers in a single layer without squeezing and the containers were closed as described above. The fruits were stored at 4°C and specimens were used at the harvest day (day 0) and on days 1, 2, 3, 5, 7, 10, 15, and 20 postharvest. The fruits of Feizixiao from the first experiment were used as control.

The pericarp must be carefully peeled from all fruit samples to avoid adhesions of pulp or juice. The pericarp samples have to be immediately frozen in LN_2_ and then stored at −70°C until use.

### Determination of the pericarp-browning index

The color of the fruits was graded based on the appearing of pericarp browning.

Grade 1: The browned area accounts for 0 to 1/4 of the total surface area of pericarp;

Grade 2: The browned area accounts for 1/4 to 1/2 of the total surface area of pericarp; Grade 3: The browned area accounts for 1/2 to 3/4 of the total surface area of pericarp; Grade 4: The browned area accounts for 3/4 to 1 of the total surface area of pericarp; and Grade 5: Completely browned.

The pericarp-browning index was calculated according to the following formula:

Pericarp-browning index  =  ([the number of fruits of Grade 1×1] + [the number of fruits of Grade 2×2] + [the number of fruits of Grade 3×3] + [the number of fruits of Grade 4×4] + [the number of fruits of Grade 5×5])/(the total number of litchi fruits surveyed ×5).

### Determination of the water loss of pericarp

The water loss of pericarp was determined, and then calculated: Water loss of pericarp (expressed in g · gDW^−1^)  =  (pericarp water content on the day of the harvest - pericarp water content on at the time of sampling)/dry weight of pericarp [17].

### Protein extraction and PPO activity assay

Two grams of pericarp samples was weighed and placed into a clean mortar, about 8 mL of phosphate buffer (0.05 mol/L, pH 7.8) and 0.2 g of insoluble polyvinylpyrrolidone were added. The pericarp was thoroughly ground in an ice bath and then centrifuged at 8000*_g_* at 4°C for 30 min. After centrifugation, the supernatant was applied as the crude enzyme extract. To determine PPO activity, total 3.0 ml reaction solution was made by adding 2.3 mL of phosphate buffer solution (PBS, 0.05 mol/L, pH 7.0), 0.5 mL of catechol (0.1 mol/L), and 0.2 mL of the crude enzyme extract, respectively. Catechol was mixed with PBS in advance, and then the crude enzyme extract was added in. The absorbance at 420 nm (A_420_) was immediately measured for 3 minutes at 25°C using a spectrophotometer. One unit of enzyme activity was defined as the increase of A_420_ by 0.1 per milligram of protein per minute [23]. The protein concentrations were determined by the dye-binding method [24].

### Genomic DNA and RNA extraction and reverse transcription

Total genomic DNA sample was extracted from young leaves of Feizixiao according to the CTAB method [25]. Total RNA was also extracted from various plant materials using a modified CTAB method [26] and then purified with the DNase I kit (TaKaRa, Dalian, China) following the manufacturer's instruction. The amount of DNA and RNA extracts were qualified and quantified using 1.0% agarose gel electrophoresis and a colorimetric assay, respectively. The first strand cDNA was synthesized using M-MLV reverse transcriptase (Invitrogen, Carlsbad, CA) with oligo (dT)_20_ as the primer. Afterwards, the samples were stored at −20°C till use.

### *LcPPO* cloning

The *LcPPO* gene fragment was amplified from genomic DNA of the Feizixiao cultivar with the degenerate primers L-PPO-F and L-PPO-R, both of them were designed corresponding to the conserved region of the *PPO* sequence (Table 1). The amplicons were sequenced, and next the sequence data were used to design the 5′- and 3′- genome walking primers L-PPO-5GW-1, L-PPO-5GW-2, and L-PPO-5GW-3 (Table 1) as well as L-PPO-3GW-1, L-PPO-3GW-2, and L-PPO-3GW-3 (Table 1). Genome-walking amplification was performed using a commercial kit following the instructions of manufacturer (TaKaRa, Dalian). The 5′- and 3′-DNA fragments of *LcPPO* were resulted and then assembled to obtain full-length *LcPPO*.

**Table 1:** Nucleotide sequences of the primers applied*.

Usage	Name	Primer sequence (5′ to 3′)
PPO degenerate primers	L-PPO-F	CCDTTCTGGAAYTGGGATTC
	L-PPO-R	CNGCNGAGTAGAAGTTSCCC
PPO cDNA primers	L-PPO-C-F	ATGTTTGCTAACCCTAAATCGC
	L-PPO-C-R	GGTATCTCCGCACCATAAGTG
5′ genome-walking primer	L-PPO-5GW-1	GCAAATGGTGTCCAACGGCAAG
	L-PPO-5GW-2	GTTCGCTTGAGAACTTGCCACATG
	L-PPO-5GW-3	CATGGGCAACTTCTACTCCGCTG
3′ genome-walking primer	L-PPO-3GW-1	AGTCTAGGAGTCTTGCCGTTGG
	L-PPO-3GW-2	AATGTTGGTGGTTGGTGATTTG
	L-PPO-3GW-3	GCCAGGAGGAGAATCCCAGTTC
5′-RACE	Lac-5′RACE-OUTER	CCAGCTCCTGGGTCCGGTTCATC
	Lac-5′RACE-INNER	CCAGAACGGCATAGCAACAGTTG
3′-RACE	Lac-3′RACE-OUTER	GTTTGCTAACCCTAAATCGCCAC
	Lac-3′RACE-INNER	GGCAAATGGTGTCCAACAGCAAG
Fluorescence-based quantitative PCR primers	RT-PPO-F	CCGCACCATAAGTGAATC
	RT-PPO-R	CACCAACCACCAACATTG
	RT-actin-F	TTGGATTCTGGTGATGGTGTG
	RT-actin-R	CAGCAAGGTCCAACCGAAG
Colony PCR primers	RV-M	GAGCGGATAACAATTTCACACAGG
	M13-47	CGCCAGGGTTTTCCCAGTCACGAC
Southern blotting primers	PPO-Southern-F	ATGTTTGCTAACCCTAAATCGC
	PPO-Southern-R	GGTATCTCCGCACCATAAGTG

*PPO degenerate primer (R = A/G, Y = C/T, M = A/C, K = G/T, S = C/G, W = A/T, H = A/C/T, B = C/G/T, V = A/C/G, D = A/G/T, N = A/C/G/T).

The coding region of *LcPPO* was predicted using the sequencing data for full-length *LcPPO* DNA. Next, the primers L-PPO-C-F and L-PPO-C-R were designed within the coding region (Table 1) for amplifying the cDNA fragment of *LcPPO*. After sequencing, the 5′-rapid amplification of cDNA ends (RACE) primers Lac-5′RACE-OUTER and Lac-5′RACE-INNER and the 3′-RACE primers Lac-3′RACE-OUTER and Lac-3′RACE-INNER were designed based on the obtained *LcPPO* cDNA sequence data (Table 1). The 5′- and 3′-cDNA fragments of *LcPPO* were obtained by 5′-RACE (5′-Full RACE Kit, TaKaRa) and 3′-RACE (3′-Full RACE Core Set Ver.2.0, Takara).

The integrity and length of the PCR amplification products were estimated by running 1.0%-agarose gel electrophoresis. The target DNA fragments were purified using a DNA gel extraction kit (TaKaRa), ligated to the pMD18-T Vector (TaKaRa), and then transformed into competent *E. coli* DH5α cells (TaKaRa). After incubation on Luria-Bertani (LB)/ampiccilin (Amp) plates for 12 h, white colonies were chosen for next experiment. Later, positive clones were found using a PCR assay with 1 μL of the culture broth as the template and the universal primers RV-M and M13-47 (Table 1). Eventually, the positive clones were also chosen and subsequently subjected to DNA sequencing.

### *LcPPO* sequence analysis

The sequence data were analyzed by using the BLASTx (http://www.ncbi.nlm.nih.gov/BLAST/) and ProtParam (http://eb.expasy.org/protparam/) tools. The open reading frame (ORF) was analyzed using ORF Finder (http://www.ncbi.nlm.nih.gov/grof/grof.htm). Sequence alignments were performed using DNAMAN 6.0 (Lynnon Corporation, Quebec, Canada). Conserved protein motifs were predicted using Sanger Pfam (http://pfam.sanger.ac.uk). The similarity of the determined amino acid sequence with the PPO amino acid sequences of other biological species was compared using MEGA 5 method [27].

### Southern blot assay of *LcPPO*

The genomic DNA of Feizixiao was digested with the restriction enzyme(s) *Eco*RIor *Xho*I and *Eco*RI. The 300 μL digestion reaction was made: 60 μg of genomic DNA, 10 μL of each restriction enzyme, 30 μL of 10 X buffer; then by adding ddH_2_O till 300 μL,Then the digestion reaction was performed at 37°C for 10–15 h. The resulted products after digestion were assessed by resolving 5-µL aliquots of the digestion samples on 0.8% agarose gels, followed by electrophoresis at 10 V · cm^−1^ for 1 h. DNA blot transfer was carried out using a semi-dry transfer cell (BIO-RAD, Hercules, CA). For preparation of southern blot probes, the primers PPO-Southern-F and PPO-Southern-R were designed corresponding to the cDNA sequence of *LcPPO* (Table 1). In the southern blot assay, probe preparation, hybridization, and membrane washing were performed with a Roche DIG-labeling kit (Roche, Shanghai) according to the manufacturer's instructions.

### *LcPPO* expression analysis

The primers RT-PPO-F and RT-PPO-R were designed according to the conserved sequence of *LcPPO*; and the primers of RT-actin-F and RT-actin-R were designed according to the known sequence of the β-actin gene (reference gene [28], [29], GenBank accession No. DQ990337.1, Table 1). The *LcPPO* and β-actin genes were simultaneously amplified from each cDNA template. The RT-PCR reaction system was used following the manufacturer's instructions (a SYBR GreeScript RT-PCR Kit II, TaKaRa, Dalian). Total 50 μL reaction system contained 25.0 μL of 2X SYBR Premix Ex Taq II, 1.0 μL of 50X ROX Reference Dye II, 4.0 μL of template (100-fold-diluted first-strand cDNA), 2.0 μL each of the up- and down-stream primers (10 μmol/L), adding ddH2O to 50 μL. The PCR cycling parameters were as follows: pre-denaturation at 95°C for 30 s, and 40 cycles of - denaturation at 95°C for 5 s followed by annealing and extension at 60°C for 34 s (with high-resolution melting analysis). After amplification, the data from the reaction plate were added to the research plate for processing. Data analyses were performed using 2^−△△CT^ method [30]–[31]. The LcPPO gene expression level in pericarp of newly-harvested Feizixiao fruit was used as the reference. All experiments were performed three times, and the arithmetic mean of replicate data were calculated and used for the comparisons.

### Data analyses

After experiment, all data were analyzed in the DPS data processing system [32]. The resulted data were presented in the text.

## Results and Discussion

### *LcPPO* cloning

A 600-bp DNA fragment was amplified from the genomic DNA of Feizixiao with the degenerate primers L-PPO-R and L-PPO-F. The fragment was purified and sequenced; new primers were designed based on the sequencing data and used for genome-walking. The resulted target sequences have 820 bps and 780 bps in length respectively. Next, the three DNA fragments were spliced to obtain the full-length DNA fragment of *LcPPO* (Figure S1).

New primers were designed within the coding region of the resulted DNA fragment, and a 600-bp cDNA fragment of *LcPPO* was amplified from the first strand cDNA reverse transcripted, using total RNA from the pericarp of Feizixiao as the template. Based on the cDNA sequence, two 1100-bp target sequences were obtained using the 3′- and 5′-RACE techniques, respectively. Then, the three cDNA sequences were spliced to obtain the full-length cDNA sequence of *LcPPO* (2120 bp, excluding the polyA tail) (Figure S1).

### *LcPPO* sequence analysis

The cDNA sequence of *LcPPO* contains a 5′-untranslated region (UTR) with 155 bp in length, a 165-bp 3′-UTR and a complete open reading frame (ORF). The ORF is 1800 bps in length and encodes a polypeptide with 599 amino acid residues. The corresponding DNA sequence of the ORF is 2015 bps in full length and contains a 215-bp intron that starts from the 842-bp position near the 5′-end (Figure S1). The deduced polypeptide has M_r_ 67,036.4 Da; its isoelectric point (pI) is 8.72 according to the analyses on ProParam. The instability parameter was 41.21, suggesting the peptide was a labile protein. The GRAVY score was −0.488, which implied that the protein was water-soluble. The Sanger Pfam online database analysis showed that the protein sequence contained three typical structural domains of PPOs, corresponding to the tyrosinase, PPO1-DWL, and PPO-KFDV domains (Figure S1).

**Figure 1:**
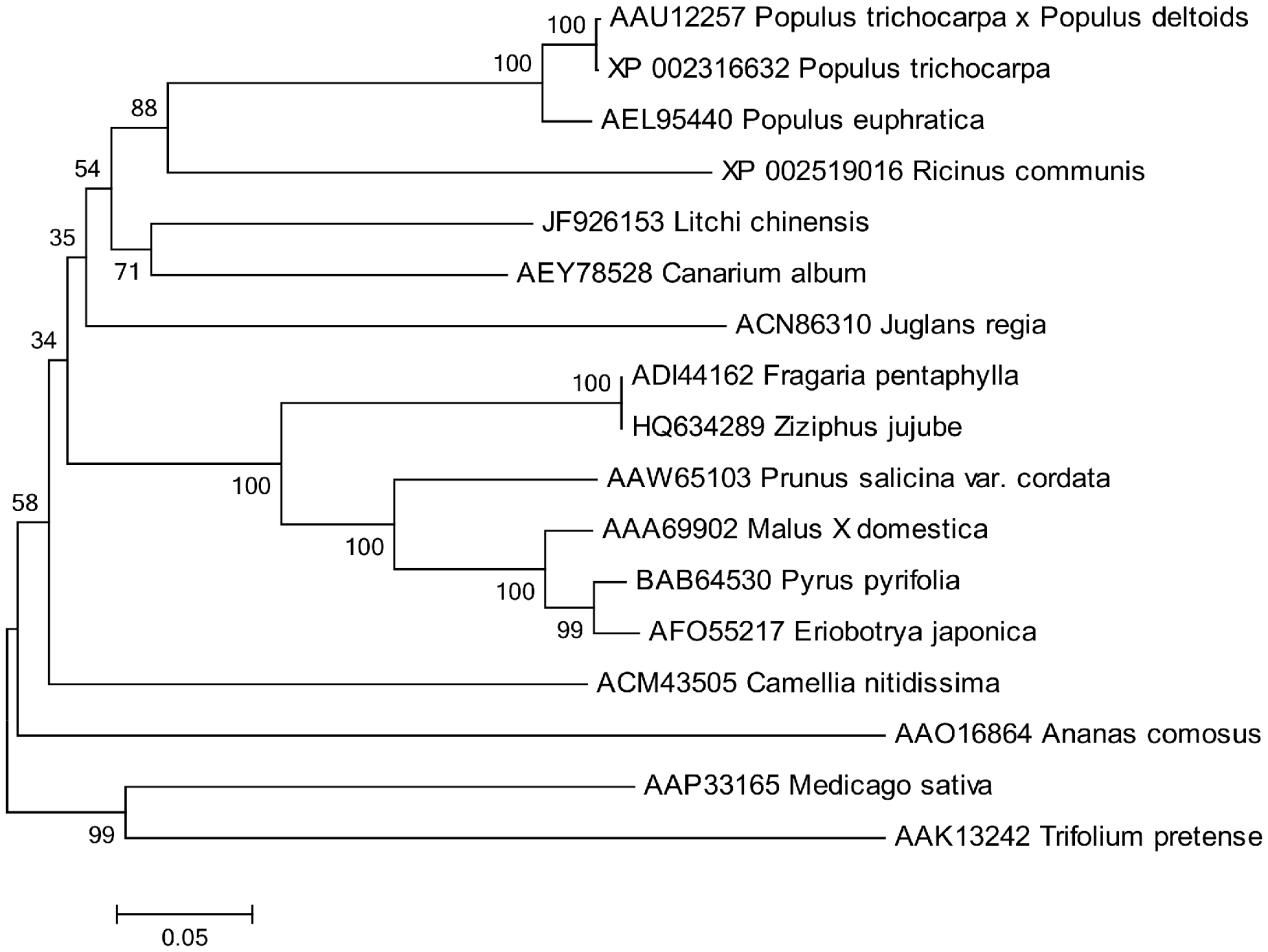
Phylogenetic tree of *LcPPO* with other PPOs from different species. All of PPO sequences except *LcPPO* (JF926153 Litchi chinensis) were from NCBI database. The polygenetic tree was drawn based on the NJ methods, which were described in Mega 5. Numbers at the branches represent percentage bootstrap support with 1000 replicates.

Compared sequence alignment, the amino acid sequence deduced from *LcPPO* cDNA shared 74% similarities to the PPO protein of *Canarium album*. A high similarity ratio (up to 68%) was observed between the deduced amino acid sequences of *LcPPO* cDNA and the PPO protein of other species, such as *Ricinus communis*, *Populus trichocarpa × Populus deltoid*, *Populus euphratica*, and *Populus trichocarpa* (Fig. 1). These results confirmed that the obtained cDNA sequence is the *LcPPO*, encoding the PPO of litchi. To our knowledge, this is the first report of the *LcPPO* sequence. The sequence has been deposited in the NCBI Genbank database under accession number JF926153.

### Southern blot assay of *LcPPO*

**Figure 2:**
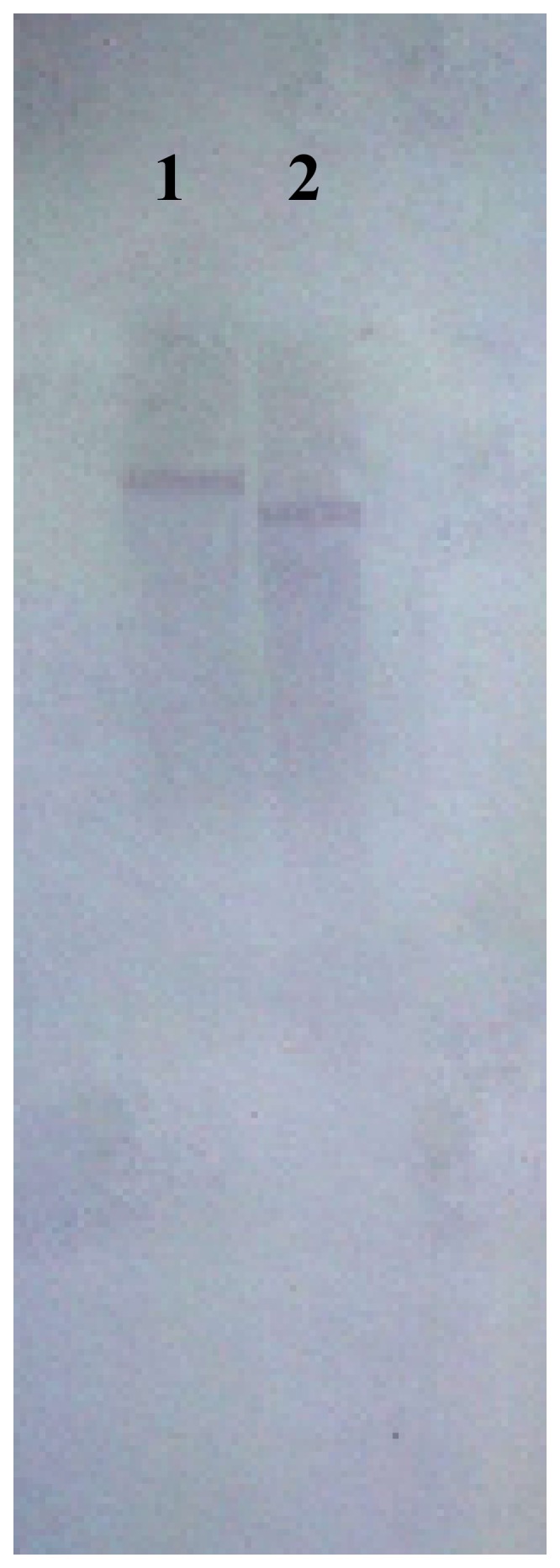
Southern blotting analysis of *LcPPO*. Numbers on the top of the lane represent that Genomic DNA from Feizixizo leaves was digested by *EcoR* I (1), and *Xho* I and *EcoR* I (2) at first, and then was hybridized with the *LcPPO*-specific probe. A clear band was visible in lane 1 and lane 2.

The genomic DNA of Feizixiao was analyzed by southern blotting. By using the *LcPPO*-specific probes, only one clear band remained visible. The molecular mass of the double-digested (*Xho*I and *Eco*RI) hybridization fragments was slightly smaller than the single-digested (*Eco*RI) hybridization fragments (Fig. 2). The *LcPPO* appeared as a single-copy gene in the litchi genome, since the designed probe sequence did not contain the *Xho*Ior *Eco*RI restriction sites.

### Tissue-specific *LcPPO* expression

**Figure 3:**
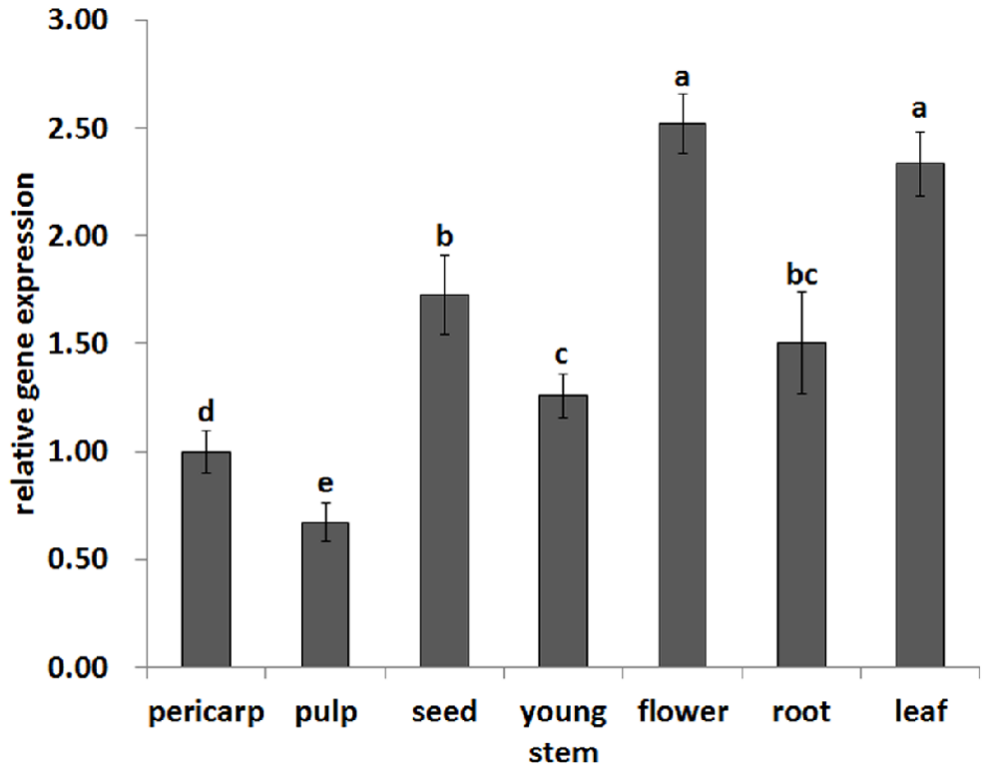
Expression of *LcPPO* in different litchi tissues of Feizixiao. The different letter above the column indicated that there were significantly different among the samples (*P*<0.05). The highest expression was found in the flower and leaf, and the lowest expression was in the pulp.

*LcPPO* was expressed in various tissues of Feizixiao, including pericarp, pulp, seed, flower, stem, leaf, and root after carrying quantitative RT-PCR assay. The *LcPPO* expression level was the highest in the flower and the leaf, followed by the seed and the root and the young stem, pericarp and pulp (the lowest; Fig. 3). The *LcPPO* expression level was significantly different (*P<0.05*) in the tissue parts, indicating that PPO may play different role.

### *LcPPO* expression in litchi pericarp at different developmental stages

**Figure 4:**
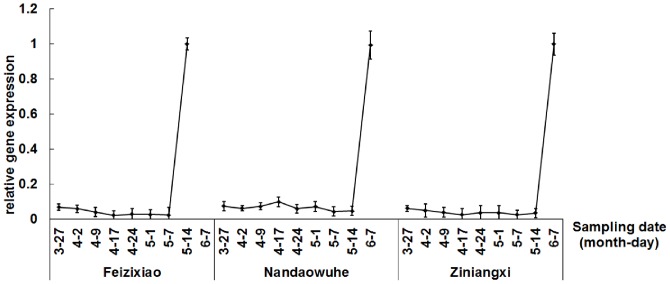
*LcPPO* expression in litchi pericarp at different developmental stages. Fruits of Feizixiao were harvested on May 14^th^, 2010; Ziniangxi and Nandaowuhe were harvested on July 7^th^, 2010. Expression of *LcPPO* in pericarp increased sharply after harvested. Similar changes were observed with Feizixiao, Ziniangxi and Nandaowuhe.

The *LcPPO* expression level of litchi pericarp was generally low and stable during fruit development, but increased rapidly after harvest. During postharvest storage, the increasing of *LcPPO* expression level in the pericarp was consistent in various cultivars, i.e., Feizixiao, Nandaowuhe, and Ziniangxi (Fig. 4), implying the *LcPPO* expression increase was induced by the harvesting process. It is noted that this is different from previous findings in apricot and banana during fruit development, which showed that PPO gene expression ceased in the color-turning stage in apricots [33] and decreases in peel of banana in the ripening stage [34]. Thus, the PPO gene expression in fruit tissues is species-specific.

### The relationship of postharvest pericarp browning with the PPO activity and *LcPPO* expression of different litchi cultivars at room temperature

**Figure 5:**
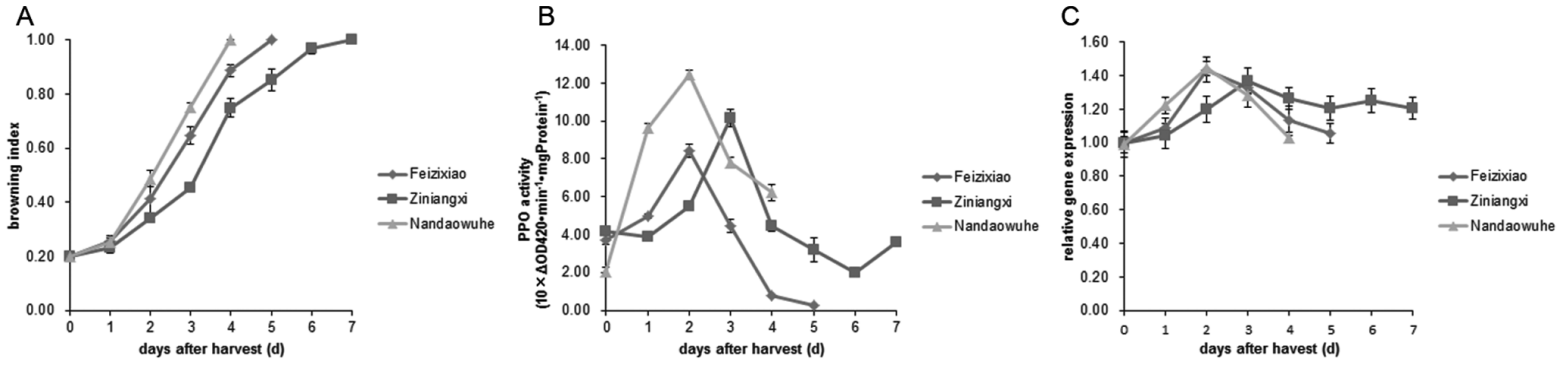
Changes in pericarp-browning index, PPO activity, and *LcPPO* expression of postharvest litchi fruits of different cultivars. Pericarp-browning index (A) of Nandaowuhe increased fast compared with Feizixiao and Ziniangxi. PPO activity (B) for Nandaowuhe was higher than Feizixiao and Ziniangxi, but *LcPPO* expression (C) level increased fast than others on the first day after harvest, and then both Nandaowuhe and Feizixiao reached highest level on day 2.

During postharvest storage, the pericarp-browning index of different litchi cultivars gradually increased over time. Overall, Nandaowuhe showed the fastest pericarp browning and turned completely browned on day 4 after harvest, whereas Feizixiao turned completely browned in day 5 after harvest. Ziniangxi showed the slowest pericarp browning and the color turned completely browned on day 7 after harvest (Fig. 5-A).The browning index values of the three litchi cultivars rapidly increased during 1–4 days after harvest. Within the same storage period, the pericarp-browning index values of Feizixiao and Nandaowuhe were constantly greater than Ziniangxi, while the values of Nandaowuhe were slightly larger than Feizixiao (Fig. 5-A).

With newly harvested litchi fruits, Nandaowuhe had the lowest pericarp PPO activity, whereas the pericarp PPO activity in Feizixiao and Ziniangxi were almost the same. During postharvest storage, the pericarp PPO activity of the three cultivars increased at the beginning, but decreased afterwards. The highest rate of increase was observed in Nandaowuhe, followed by Feizixiao, and Ziniangxi. The pericarp PPO activity reached the peaks in Feizixiao and Nandaowuhe on day 2 after harvest, however the enzyme activity was lower in the former cultivar than in the latter. In Ziniangxi, it appeared as a peak on day 3 after harvest. Within the same storage period, Nandaowuhe had the highest pericarp PPO activity on day 2 after harvest, followed by Feizixiao and Ziniangxi (Fig. 5-B).

The *LcPPO* expression level in litchi pericarp showed similar variations to the PPO activity. Within 2 d after harvest, Nandaowuhe exhibited the highest pericarp *LcPPO* expression level, followed by Feizixiao, and Ziniangxi. The pericarp *LcPPO* expression level of Ziniangxi maintained stable from 3 d to 7 d postharvest storage (Fig. 5-C).

The differences in postharvest pericarp browning of various litchi cultivars are related to the pericarp PPO activity at an early stage of storage. In addition, the level of pericarp PPO activity in various litchi cultivars at the early stage of storage is consistent with the corresponding *LcPPO* expression levels, the PPO activities and *LcPPO* expression levels show similar variations. These findings demonstrate that the differences in the pericarp browning process among various litchi cultivars are positively correlated with the *LcPPO* expression level at the early stage of postharvest storage. It is possible that the differences in the *LcPPO* expression level among various cultivars determine the differences in the corresponding PPO activity, resulting in the different levels of pericarp browning in different cultivars.

### The effect of water loss control on litchi pericarp browning and PPO activity and *LcPPO* expression

**Figure 6:**
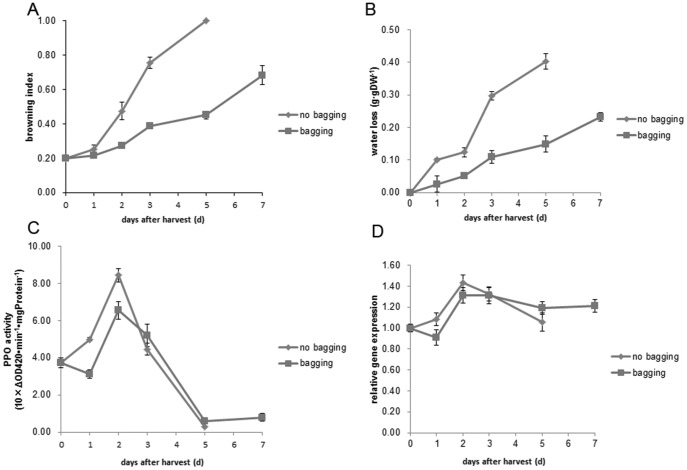
Changes in pericarp-browning index, pericarp water loss, PPO activity, and *LcPPO* expression of Feizixiao fruit under packaging treatment. A. Pericarp-browning index: B. Pericarp water loss; C. PPO activity; and D. *LcPPO* expression. Fruit bagging reduced the water loss in pericarp of Fizixiao, and slowed down the pericarp-browning. During the first three days of storage, both the PPO activity and the *LcPPO* expression were decreased in bagged fruit.

Postharvest storage of litchi fruits in 0.2-μm preserving package delayed pericarp browning (Fig. 6-A) and reduced the pericarp water loss rate (Fig. 6-B). These results are consistent with others [35]. The pericarp PPO activity and the *LcPPO* expression level of litchi fruits under packaging treatment and the control treatment were all increased at the first, and then decreased, with the peak values observed on day 2 after harvest. The packaging treatment substantially decreased the pericarp PPO activity and *LcPPO* expression level 3 d after harvest, but had no significant effects on the two parameters thereafter (Fig. 6-C, -D).

The practices such as packaging treatment [35] and pericarp coating [36] can prevent postharvest pericarp water loss and delay the pericarp browning of fruits. These measures possibly reduce the pericarp *LcPPO* expression at the early stage of postharvest storage, thereby reducing the pericarp PPO activity and then suppressing the related enzymatic reaction.

### The effect of temperature on litchi pericarp browning and PPO activity and *LcPPO* expression

**Figure 7:**
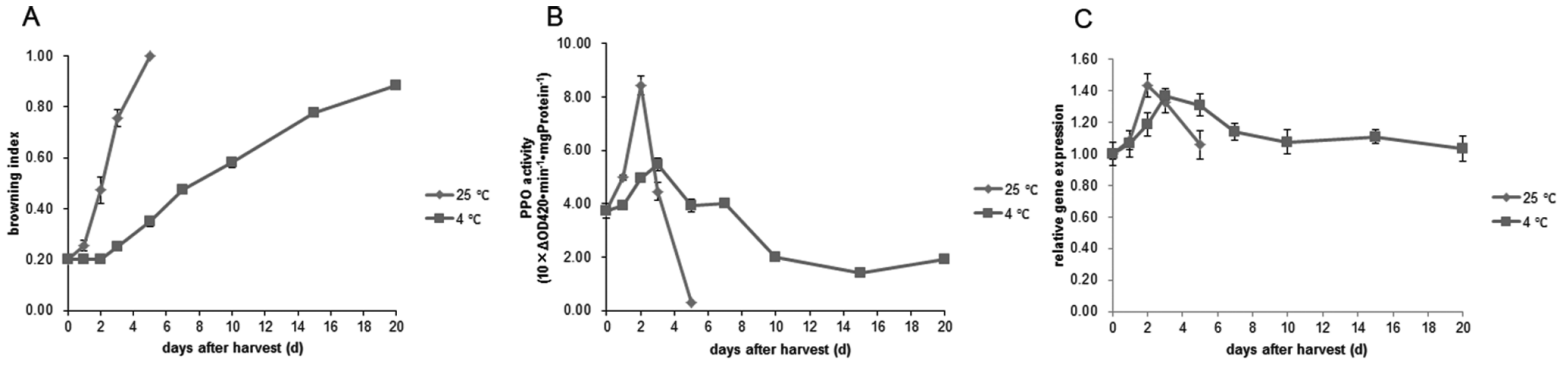
Changes in pericarp-browning index, PPO activity, and *LcPPO* expression of Feizixiao fruit after stored at the low temperature. A. Pericarp-browning index: B. PPO activity, and C. *LcPPO* expression Pericarp browning was delayed while stored the fruit at 4°C. The low temperature also can cause the reduction of the PPO activity and *LcPPO* expression level in pericarp during the first 2 days after harvest.

When stored at 4°C, the postharvest pericarp browning of litchi fruit slowed down and the pericarp-browning index was only 0.8 on day 20 after harvest, whereas the color of control fruits had turned completely brown by day 5 after harvest (Fig. 7-A).

The low-temperature storage reduced the pericarp PPO activity within 2 d after harvest, but the pericarp PPO activity of fruits at different temperatures normally exhibited an increase followed by a decrease. The pericarp PPO activity showed the increase quickly at room temperature resulting forming a peak on day 2 after harvest. At low temperature, the pericarp PPO activity peaked on day 3 after harvest and the peak value was around half of that at room temperature. After the peak forming time, the pericarp PPO activity rapidly decreased at room temperature, but exhibited a decrease slowly at low temperature (Fig. 7-B).

Within days 1–2 d after harvest, the pericarp *LcPPO* expression level was lower in fruits stored at low temperature than stored at room temperature, indicating that *LcPPO* expression was inhibited by low temperature (Fig. 7-C). Our finding of *LcPPO* expression in litchi fruit is different from previous record in pineapple fruit [37]. The PPO gene expression in postharvest pineapple fruit could be induced quickly by low temperature, possibly causing the occurrence of blackheart [37]. Under treatment with either room temperature or low temperature, the pericarp *LcPPO* expression level showed increases followed by decreases. The expression level of litchi fruits at room temperature peaked on day 2 after harvest, while that of fruits at low temperature peaked on day 3 after harvest. The peak values of pericarp *LcPPO* expression level coincided with those of the PPO activity, and the pericarp *LcPPO* expression level was lower at low temperature than at room temperature. The *LcPPO* expression level decreased gradually regardless of the temperature after the peak time (Fig. 7-C).

The above results suggest that low temperature can suppress pericarp *LcPPO* expression at the early stage of postharvest storage, and later it would reduce the PPO activity. This may be one of the main reasons for the delaying of postharvest pericarp browning at low temperatures.

In conclusion, we obtained the cDNA (JF916253) and DNA sequences of *LcPPO*. Our experimental data showed that the *LcPPO* gene is expressed in various litchi tissues with the different level, such as the highest expression was found in litchi flower and leaf. At an early stage of the postharvest period, the pericarp *LcPPO* expression level is relatively low in peripcarp. The expression of *LcPPO* is induced by pericarp water loss and suppressed by low temperature. During storage, the pericarp *LcPPO* expression level and PPO activity had similar variations and positive correlation (*r* = 0.5708, *P*<0.001). Furthermore, the increases in the *LcPPO* expression level and PPO activity in pericarp were consistent with those in the pericarp-browning index values at the early stage of postharvest storage. Therefore, the up-regulation of *LcPPO* expression at the early stage of postharvest storage likely accelerates PPO protein synthesis. As a consequence, it may enhance the PPO activity at the early stage; a further accelerating browning of litchi pericarp could be happen. Our results provide some solid supporting data for the development of strategies for better controlling the postharvest pericarp browning of litchi fruit, e.g., by breeding of long-storage litchi cultivars.

## Supporting Information

Figure S1Genomic DNA, cDNA, and deduced amino acid residue sequences of *LcPPO*. DNA: genome sequence; cDNA: cDNA sequence; AA: deduced amino acid residue sequences; the underlined sections indicate the conserved domains and the introns;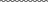: Tyrosinase domain;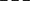: PPO1-DWL domain,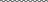: PPO1-KFDV domain;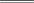: intronic sequences.(TIF)
